# Tumorigenesis of Papillary Thyroid Cancer Is Not *BRAF*-Dependent in Patients with Acromegaly

**DOI:** 10.1371/journal.pone.0110241

**Published:** 2014-10-17

**Authors:** Hee Kyung Kim, Ji Shin Lee, Min Ho Park, Jin Seong Cho, Jee Hee Yoon, Soo Jeong Kim, Ho-Cheol Kang

**Affiliations:** 1 Department of Internal Medicine, Chonnam National University Medical School, Gwangju, Korea; 2 Department of Pathology, Chonnam National University Medical School, Gwangju, Korea; 3 Department of Surgery, Chonnam National University Medical School, Gwangju, Korea; Catalan Institute of Oncology, Spain

## Abstract

**Introduction:**

Several studies have reported a high frequency of papillary thyroid cancer (PTC) in patients with acromegaly. The aim of this study was to determine the prevalence and predictors of thyroid cancer in patients with acromegaly and to investigate the frequency of the *BRAF* ^V600E^ mutation in PTC patients with and without acromegaly.

**Materials and Methods:**

We conducted a retrospective study of 60 patients with acromegaly. Thyroid ultrasonography (US) and US-guided fine needle aspiration were performed on nodules with sonographic features of malignancy. We selected 16 patients with non-acromegalic PTC as a control group. The *BRAF* ^V600E^ mutation was analyzed in paraffin-embedded surgical specimens of PTC by real-time polymerase chain reaction, and tumor specimens from patients with PTC were stained immunohistochemically with an antibody against insulin-like growth factor-1 receptor β (IGF-1Rβ).

**Results:**

Thyroid cancer was found in 15 (25.0%) patients. No differences in age, sex, initial growth hormone (GH) and IGF-1 percentage of the upper limit of normal values or treatment modalities were observed between patients with and without PTC. Acromegaly was active in 12 of 15 patients at the time of PTC diagnosis; uncontrolled acromegaly had a significantly higher frequency in the PTC group (60%) than in the non-PTC group (28.9%) (*p* = 0.030). The *BRAF* ^V600E^ mutation was present in only 9.1% (1/11) of PTC patients with acromegaly, although 62.5% (10/16) of control patients with PTC had the mutation (*p* = 0.007). IGF-1Rβ immunostaining showed moderate-to-strong staining in all malignant PTC cells in patients with and without acromegaly. Significantly less staining for IGF-1Rβ was observed in normal adjacent thyroid tissues of PTC patients with acromegaly compared with those without (*p* = 0.014).

**Conclusion:**

The prevalence of PTC in acromegalic patients was high (25%). An uncontrolled hyperactive GH-IGF-1 axis may play a dominant role in the development of PTC rather than the *BRAF* ^V600E^ mutation in patients with acromegaly.

## Introduction

Acromegaly is a chronic disease resulting from excessive secretion of growth hormone (GH) and insulin-like growth factor-1 (IGF-1). IGF-1 promotes mitosis and suppresses apoptosis of cells by binding to the IGF-1 receptor β (IGF-1Rβ), and is thought to be responsible for the increased risk of developing malignancies, mainly colorectal, breast, prostate, and hematologic [1], [2].

Several studies have reported a high frequency of thyroid cancer mostly papillary thyroid cancer (PTC) in patients with acromegaly. The reported prevalence is 4.7–11%, which is much higher than that in the general population [3]–[6]. However, the actual incidence of thyroid cancer in patients with acromegaly and the impact of active acromegaly on the development of thyroid cancer is unknown due to the relative rarity of the condition [7].

Recent studies have reported that the point mutation in *BRAF* is frequently detected in PTC patients [8], and the prevalence of the *BRAF* ^V600E^ mutation is higher in Korea (50–83%) than in Western countries [9]–[11]. The *BRAF* ^V600E^ mutation has been shown to cause continuous and uncontrolled activation of the kinase pathway, and it is associated with a poor prognosis for PTC [12]. However, it is not known whether the *BRAF* mutation is associated with PTC in patients with acromegaly.

The aim of this study was to determine the prevalence and predictors of thyroid cancer in patients with acromegaly and to investigate the frequency of the *BRAF* ^V600E^ mutation in PTC patients with and without acromegaly.

## Patients and Methods

### Patients

Thirty newly diagnosed patients with acromegaly were referred to Chonnam National University Hwasun Hospital between April 2004 and April 2013. Except for two patients who presented with inoperable tumors, the patients (n = 28) underwent pituitary surgery in our center. In addition, 30 patients who had been previously treated for acromegaly were referred for postoperative follow-up during the same time period. Thus, 60 patients were retrospectively reviewed, and clinical parameters associated with acromegaly, including age at diagnosis, secreting type of tumor, treatment modality, other co-morbid diseases, and status of disease during follow up were examined. Thyroid ultrasonographic (US) images and reports were also reviewed. The diagnosis of acromegaly and definition of active disease were based on clinical features, lack of GH suppression <1.0 ng/mL after a 75 g oral glucose load, and elevated fasting IGF-1 level (above the age- and sex-matched reference range) [13]. IGF-1 levels were expressed as percentages of the upper limit of age-adjusted normal levels (% ULN). We randomly selected 16 patients with non-acromegalic PTC who underwent thyroid surgery at our hospital between May and August 2010 as a control. This study protocol was reviewed and approved by the Institutional Review Board of the Chonnam National University Hwasun Hospital, Hwasun, Korea. Written informed consent was obtained from all participants.

### Thyroid US and US-guided fine needle aspiration cytology (US-FNAC)

Thyroid US was performed using a 10–13 MHz linear probe (Logiq9, GE Medical Systems, Milwaukee, WI, USA or ACUSON Antares, Siemens Medical Solutions, Malvern, PA, USA) by a single endocrinologist. US-FNAC was performed on thyroid nodules >1 cm in diameter or on nodules presenting one of the sonographic features of malignancy, including those with marked hypoechogenicity, micro- or macro-calcifications, a taller-than-wide in shape, or spiculated margins [14] regardless of size.

**Figure 1:**
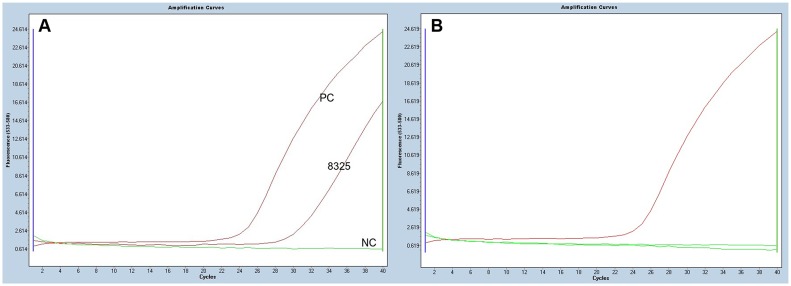
Real-time qPCR analysis for the *BRAF* ^V600E^ mutation. A. Mutation positive. B. Wild type.

### Biochemical measurements

All blood samples were collected after an overnight fast. Serum GH (normal range, 0–10 ng/mL) and IGF-1 levels were measured using an immunoradiometric assay (HGH-CTK IRMA, Diasorin, Sallugia, Italy) and a chemiluminescence immunoassay (CLIA, IGF-1 Immulite, DPC, Los Angeles, CA, USA), respectively.

### DNA isolation and detection of the *BRAF* ^V600E^ mutation

Thyroid cancer specimens were provided by the Chonnam National University Hwasun Hospital National Biobank of Korea, a member of the National Biobank of Korea, which is supported by the Ministry of Health, Welfare and Family Affairs. A 10-µm paraffin-embedded section was obtained from each sample from patients with and without acromegaly and subjected to genomic DNA extraction using the QIAamp DNA Minikit (Qiagen, Chatsworth, CA, USA) according to the manufacturer’s instructions. Real-time PCR was performed using the LightCycler 480 (Roche Diagnostics, Indianapolis, IN, USA) under the following conditions: one cycle of 2 min at 50°C, followed by 10 min at 95°C for one cycle, then 40 cycles of 15 s at 95°C, and finally 45 s at 60°C. The Real-Q BRAF V600E Detection kit (Biosewoom, Seoul, Korea) was used for the PCR reaction. The Real-Q BRAF Detection Kit is a ready-to-use kit for the detection of the *BRAF* ^V600E^ (1799T>A) somatic mutation of the *BRAF* oncogene in a background of wild-type genomic DNA using a multiplex real-time PCR assay based on the TaqMan MGB probe system. The kit supplies two assays. The *BRAF* mutation assay is labeled with VIC (define acronym), and contains an allele specific forward primer for the discrimination of the V600E mutation. The internal control assay, labeled with 6-carboxyfluorescein (FAM), is used to assess nucleic acid isolation and possible PCR inhibition. The kit amplifies a region in exon 8 of the *BRAF* gene. The primer and probe are designed to avoid the *BRAF* polymorphisms. For clinical samples, the presence of the BRAF V600E mutation was determined using the instructions for the Real-Q BRAF V600E Detection Kit. The cycle threshold (Ct) for RQ PCR was defined as the cycle at which a significant increase in fluorescence was detected. If the FAM signal (control assay) was observed simultaneously, then ΔCt values were calculated by subtracting the control Ct value from the mutation Ct value. Samples with ΔCt over 13 cycles were considered negative for the *BRAF* ^V600E^ mutation according to instructions for the Real-Q BRAF V600E Detection Kit.

### Immunohistochemistry

Thyroid cancer specimens were selected based on a histological analysis by a pathologist. Normal thyroid tissues were taken from histologically normal areas adjacent to thyroid cancers. Automated immunohistochemical staining was performed using the Bond-max system (Leica Microsystems, Bannockburn, IL, USA), which can process up to 30 slides at a time. Slides carrying the tissue sections cut from paraffin-embedded tissue blocks were labeled and dried for 1 h at 60°C. These slides were then covered by Bond Universal Covertiles (Leica Microsystems) and placed into the Bond-max instrument. All subsequent steps were performed by the instrument according to the manufacturer’s instructions (Leica Microsystems). The antibody used was a rabbit polyclonal antibody against human IGF-IRβ (1∶1200, Cell Signaling Technology, Danvers, MA, USA). The antigen–antibody complex was visualized using diaminobenzidine as the chromogen. Slides were counterstained with Mayer’s hematoxylin, washed in fresh water, dehydrated, and mounted. We used a semi-quantitative approach to evaluate IGF-1Rβ, based on staining intensity (SI) and percentage of positive cells (PP), to create an immune-reactive score (IRS) as follows: IRS = SI×PP as described previously [15]. Staining intensity was scored as follows: 0 = no staining, 1 = weakly positive, 2 = moderately positive, and 3 = strongly positive. Scoring of the staining pattern was based on the percentage of positive tumor cells: 0 = 0–5%, 1 = 6–25%, 2 = 26–50%, 3 = 51–100%. Thus, the IRS score ranged from 0 to 9 (0 was Grade 0, 1–3 were Grade 1, 4–6 were Grade 2, and 7–9 were Grade 3).

### Statistical analysis

Differences in non-categorical and categorical factors between the patients with and without thyroid cancer were compared using the Mann–Whitney *U*-test and χ^2^ or Fisher’s exact test, respectively. All statistical analyses were performed using SPSS 17.0 (SPSS Inc., Chicago, IL, USA). A value of p<0.05 was taken to indicate statistical significance.

## Results

### Clinical details of the study population

Clinical characteristics and coexisting malignancies of the 60 patients are described in Table 1. Fifty-eight patients underwent pituitary surgery, and a somatostatin analogue or radiotherapy was added as adjuvant treatment in 27 and 10 patients, respectively. During the follow-up period (mean, 84.8 months), 22 patients (36.7%) showed continued evidence of uncontrolled acromegaly despite additional medical treatment. Malignancy was found in 21 patients (35.0%). PTC was found in 15 patients (25.0%), gastric cancer in one (1.7%), colon cancer in five (8.3%), breast cancer in two (3.3%), renal cell cancer in one (1.7%), endometrial cancer in one (1.7%), and pancreatic cancer in one (1.7%) (Table 1). In patients with PTC, five patients had other cancers, including renal cell cancer, endometrial cancer, pancreatic cancer and two with colon cancer.

**Table 1:** Clinical characteristics of 60 patients with acromegaly.

Sex, n (%)	
Female	33 (55.0)
Age at diagnosis, years (range)	45.3±14.4 (16–74)
Etiology of acromegaly, n (%)	
Pituitary microadenoma/macroadenoma	7/53 (11.7/88.3)
Treatment of acromegaly, n (%)	
Surgery only	30 (50.0)
Surgery + medical treatment	18 (30.0)
Surgery + medical treatment + radiotherapy	9 (15.0)
Surgical + radiotherapy	1 (1.7)
Medical treatment only	2 (3.3)
Secreting type (n = 49)*, n (%)	
Growth hormone only	19 (38.8)
Growth hormone + prolactin	19 (38.8)
Growth hormone + other pituitary hormone	11 (22.4)
Colonoscopy (n = 48), n (%)	
Colon cancer	5 (10.4)
Tubular adenoma	14 (29.2)
Hyperplastic polyp	16 (33.3)
No polyp	13 (27.1)
Malignancy, n (%)	
All malignancy	21 (35.0)
Only papillary thyroid cancer	10
PTC with other cancer¶	5
Gastric cancer	1
Colon cancer	3
Breast cancer	2
Follow-up periods, months (range)	84.8±75.4 (0–341)
Uncontrolled acromegaly, n (%)	22 (36.7)

All scale data are means ± standard deviation.

PTC, papillary thyroid cancer.

*Nine patients failed to provide data for secreting type of pituitary adenoma because of operations in other hospitals.

¶ Five patients with PTC also had other cancers, including renal cell cancer, endometrial cancer, pancreatic cancer, and two with colon cancer.

### Thyroid evaluation

All patients underwent thyroid US, except three who had a history of thyroidectomy for PTC at other hospitals. Forty-two of the remaining 57 patients had thyroid nodules (nine had solitary, 33 had multiple), giving a point prevalence of 75.0% (45/60), including the patients who underwent thyroidectomies. US-FNAC was performed in 36 patients with thyroid nodules; four had inadequate samples, 20 had benign cytology, and 12 had PTC. No results of atypia of undetermined significance or follicular neoplasm were observed. The inadequate aspirated nodules were a homogeneous isoechoic pattern, which was consistent with a benign nature; thus, no additional FNAC was implemented. All patients with PTC on cytology underwent thyroidectomy (11 in our hospital and one at an outside hospital), and histological examinations revealed PTC in all cases. Among them, one patient had both papillary and follicular cancer. The tumors were 0.2–2.1 cm in size, and 58.3% (7/12) of PTC were micropapillary thyroid cancer. Tumor stages were 10 patients in stage I, and two patients in stage III (Table 2).

**Table 2:** Clinical characteristics, treatment, and outcomes of 15 patients with thyroid cancer.

No	Sex	Age*	Other diseases	Treatment foracromegaly	Active acromegalyat diagnosis ofthyroid cancer	Thyroid cancer						
						Age†	Histology	Tumor size (cm)	Stage	Treatment	*BRAF* ^V600E^mutation	FUmonths
1	F	72	DM, Colonpolyps	Medical	Yes	73	Papillary	0.8	I	TT	No	38
2	M	44	Colon polyps	Op + Medical	No	47	Papillary	0.9	I	RTL	No	98
3	F	51	Colon cancer	Op	Yes	51	Pap-Fol	1.0	I	TT + RAI	No	163
4	F	16	DM	Op	Yes	33	Papillary	0.8	I	TT + RAI	No	39
5	M	48	DM, Colonpolyps	Op	Yes	50	Papillary	0.2	I	TT	No	40
6	F	31	Colon polyps	Op + Medical + RT	No	55	Papillary	1.8	III	TT + RAI	No	57
7	F	17	DM, Colonpolyps	Op + Medical + RT	Yes	39	Papillary	1.1	I	TT	No	83
8	F	49	DM, Endometrialcancer	Op + Medical	Yes	49	Papillary	1.5	I	TT	No	105
9	M	29	None	Op + Medical	Yes	29	Papillary	0.7	I	TT	No	34
10	M	59	Colon cancer	Medical	No	60	Papillary	0.6	III	TT + RAI	Detected	25
11	M	34	None	Op	Yes	34	Papillary	2.1	I	TT + RAI	No	4
12¶	F	54	Colon polyp	Op	Yes	62	Papillary	0.4	I	TT	Not tested	6
13¶	F	69	DM	Op	Yes	69	Papillary	UA	UA	TT	Not tested	246
14¶	F	40	RCC, Colonpolyp	Op + Medical	Yes	40	Papillary	UA	UA	TT	Not tested	166
15¶	F	58	Pancreascancer	Op	Yes	58	Papillary	UA	UA	TT + RAI	Not tested	147

F, female; M, male; DM, diabetes mellitus; RCC, renal cell cancer; Op, operation; RT, radiotherapy; Pap-Fol, papillary-follicular mixed type carcinoma; TT, total thyroidectomy; RTL, right thyroid lobectomy; RAI, radioactive iodine ablation; UA, unavailable; FU, follow up.

*Age at diagnosis of acromegaly.

† Age at diagnosis of thyroid cancer.

¶ Patients who underwent thyroidectomies at other hospitals.

### Clinical comparisons of patients with and without thyroid cancer

Including patients who underwent thyroidectomies at other hospitals, 25.0% of all patients (15/60) were diagnosed with PTC. Thyroid US was performed in 37 patients (61.7%) at the acromegaly diagnosis, and the remaining 23 patients underwent US 8.2±6.5 years (range, 1–23.7 years) after the acromegaly diagnosis. No significant differences in age, sex, treatment modality, or GH or IGF-1 levels at the initial diagnosis of acromegaly were observed between patients with PTC and those without (Table 3). In 23 patients who underwent delayed US, uncontrolled acromegaly occurred in 85.7% (6/7) of patients in the PTC group, and 37.5% of patients (6/16) in the non-PTC group (*p* = 0.045). At the time of last follow up, the PTC group showed a significantly higher prevalence of uncontrolled acromegaly than that in the non-PTC group (*p* = 0.030) (Table 3). Acromegaly was active in 12 of the 15 patients at the time of PTC diagnosis (Table 2). After a mean follow-up of 83.4±70.2 months (range, 4–246 months), all patients were alive and PTC-free. The characteristics of the 15 patients with PTC are summarized in Table 2.

**Table 3:** Clinical comparisons of acromegalic patients with and without thyroid cancer.

Variable	Thyroid cancer		*P*
	No (n = 45)	Yes (n = 15)	
Female sex (n, %)	23 (51.1)	10 (66.7)	0.294
Age at diagnosis of acromegaly (years)	45.5±13.6	44.7±17.0	0.862
Diabetes (n, %)	16 (35.6)	6 (40.0)	0.757
Initial laboratory findings (mean ± SD)			
GH, ng/mL	36.9±51.0	32.7±20.4	0.794
IGF-1, % ULN	293.9±114.8	365.6±179.7	0.256
Surgery for pituitary mass (n, %)	44 (97.8)	14 (93.3)	0.409
Medical treatment for acromegaly (n, %)	20 (44.4)	9 (60.0)	0.296
Radiation for pituitary mass (n, %)	8 (17.8%)	2 (13.3%)	0.689
US performed at diagnoses of acromegaly (n, %)	29 (64.4)	8 (53.3)	0.443
Laboratory finding when performed US			
GH, ng/mL	25.5±48.9	11.3±13.5	0.326
IGF-1, % ULN	235.6±132.5	220.7±164.6	0.745
Uncontrolled acromegaly (n, %)	13 (28.9)	9 (60.0)	0.030

All scale data are means ± standard deviation (SD).

US, ultrasonography; GH, growth hormone; IGF-1, insulin-like growth factor-1; % ULN, percentages of the upper limit of age-adjusted normal levels.

### RQ PCR analysis for the *BRAF* ^V600E^ mutation (Figure 1)

Among 11 nodules histologically confirmed as PTC in our hospital, one (9.1%) was positive for the *BRAF* ^V600E^ mutation. The patient with the *BRAF* ^V600E^ mutation that initially presented with memory impairment and hyperthyroidism was subsequently diagnosed (increased free T4 levels with inappropriately increased TSH levels). The patient was diagnosed with a TSH-secreting adenoma and GH excess. In the non-acromegalic patients with PTC as a control group, 62.5% (10/16) of nodules had the *BRAF* ^V600E^ mutation, which was significantly higher than acromegalic patients with PTC (*p* = 0.007) (Table 4).

**Table 4:** Clinical comparisons of PTC patients with and without acromegaly.

Variable	Acromegaly		*P*
	Yes (n = 11)	No (n = 16)	
Female sex (n, %)	6 (54.5)	14 (87.5)	0.071
Age at diagnosis of PTC, years	47.3±13.0	51.6±11.8	0.381
Tumor size, cm (mean ± SD)	0.94±0.45	0.99±0.81	0.849
Multiplicity (n, %)	3 (30.0)	6 (37.5)	0.517
LN metastasis (N0/N1a/N1b/Nx)	7/3/0/1	6/5/1/4	0.279
Extrathyroidal extension (n, %)	1 (9.1)	1 (6.3)	0.600
*BRAF* ^V600E^ mutation (n, %)	1 (9.1)	10 (62.5)	0.007
IRS score of IGF-1Rβ IHC (mean ± SD)†			
Tumor tissue	3.9±1.4	4.3±1.9	0.561
Adjacent normal tissue	0.3±0.9	2.4±2.3	0.014
Grade of IGF-1Rβ IHC (n)†			
Tumor tissue (0/1/2/3)	0/7/3/0	0/10/5/1	0.711
Adjacent normal tissue (0/1/2/3)	9/1/0/0	4/8/4/0	0.005

PTC, papillary thyroid cancer; LN, lymph node; IHC, immunohistochemical; SD, standard deviation; IRS, immune-reactive score.

† One patient with acromegalic PTC could not undergo immunostaining for IGF-1Rβ.

### Immunohistochemical staining for IGF-1Rβ

**Figure 2:**
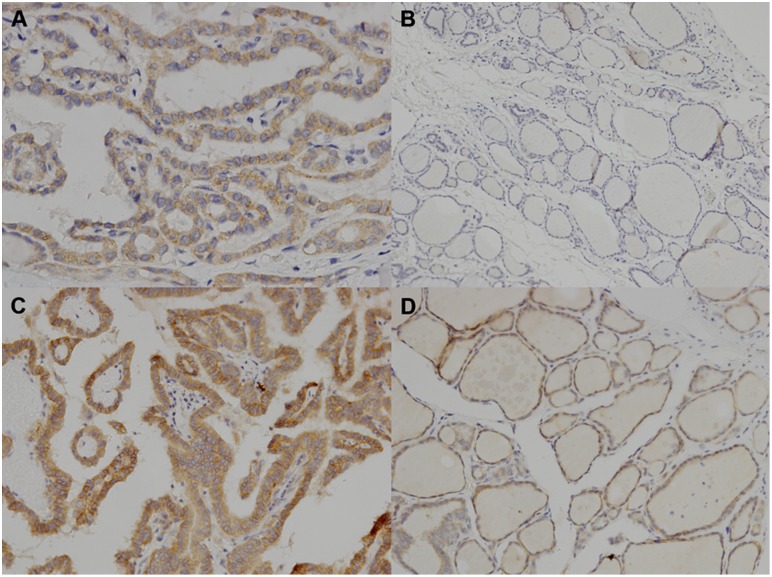
Illustrative examples of immunohistochemical expression and immunoreactive score (IRS) evaluation. A. Grade 2 insulin-like growth factor-1 receptor (IGF-1Rβ) staining in 100% of cancer cells (IRS = 6) in patients with acromegaly (×400); B. IGF-1Rβ staining absent in adjacent normal tissue (IRS = 0) in the same patients with acromegaly as in A (×100); C. Grade 3 IGF-1Rβ staining in 100% of cancer cells (IRS = 9) in patients without acromegaly (×200); D. Grade 2 IGF-1Rβ staining in 100% of adjacent normal tissue (IRS = 6) in the same patients without acromegaly as in C (×200).

IGF-1Rβ immunohistochemical staining results were obtained from 10 acromegalic PTC samples and 16 non-acromegalic PTC samples. Immunohistochemical staining of tumor tissue had a significantly higher IRS compared with the normal adjacent tissue in both groups (acromgalic group, *p*<0.001 and non-acromegalic group, *p* = 0.015). The pattern of IGF-1Rβ immunostaining was moderate to strong (IRS≥3) in all tumor cases in both the acromegalic and non-acromegalic groups (Figure 2A and C). No difference in the IRS of IGF-1Rβ expression was observed between patients with and without acromegaly (Table 4). In contrast, immunohistochemical staining in adjacent normal tissue showed a significantly lower IRS in patients with acromegaly compared with those without (*p* = 0.014). Adjacent normal tissue was grade 0 in 90% (9/10) of cases with acromegaly (Figure 2B) and 25.0% (4/16) of cases without acromegaly. A grade of 2 or 3 in adjacent normal tissue was not observed in cases with acromegaly but was found in four cases (25%) without acromegaly (Figure 2D).

## Discussion

We found thyroid nodules and cancers in 75.0% (45/60) and 25.0% (15/60) of patients with acromegaly, respectively. This prevalence of thyroid cancer is higher than that in the general population (2.5% in Korea) [16]. Uncontrolled acromegaly was significantly more frequent in the PTC group than in the non-PTC group. Only one patient (9.1%) with PTCs was positive for the *BRAF* ^V600E^ mutation. IGF-1Rβ was strongly expressed in tumor tissue but expression was lower in adjacent normal tissue. An uncontrolled hyperactive GH-IGF-1 axis rather than the *BRAF* ^V600E^ mutation may play a dominant role in the development of PTC in patients with acromegaly. This is the first report of the prevalence of the *BRAF* ^V600E^ mutation and the IGF-1Rβ staining pattern in patients with acromegalic PTC.

The thyroid gland is the most common site of all primary endocrine cancers globally [17] and is now the most common cancer in Korean females [18]. The *BRAF* isoform of *RAF* has been implicated in the pathogenesis of PTC, and *RAF* proteins are serine-threonine kinases that activate the *RAF/MEK/MAPK* signaling pathway. The T1799A mutation of the *BRAF* gene, which was originally found in >50% of malignant melanomas and a smaller percentage of colon cancers, occurs in 50–83% of PTC in Korea, where iodine consumption is very high [9]–[11]. The *BRAF* ^V600E^ mutation initiates thyroid follicular cell transformation, inducing oncogenesis in PTC with frequent local invasion and is therefore regarded as a poor prognostic factor and a diagnostic marker for PTC [12]. In our study, prevalence of the *BRAF* ^V600E^ mutation in patients with non-acromegalic PTC was 62.5%, which was similar to other Korean reports. However, only one patient (9.1%) with acromegalic PTC had the *BRAF* mutation, which was a significantly lower frequency than that in PTC patients without acromegaly. Therefore, the *BRAF* ^V600E^ mutation may not be the main mechanism of PTC carcinogenesis in patients with acromegaly.

Although controversy remains regarding general cancer risk [19], higher rates of colon cancer in patients with acromegaly compared to the general population have been reported [20], [21]. IGF-1 exerts anti-apoptotic effects and promotes epithelial proliferation, which is an important first step in the pathway to adenoma formation. An increased risk of premalignant colonic polyps at a rate of 24–38% has been reported, and strong evidence exists for an increased risk of colorectal cancer in patients with acromegaly; therefore, screening by colonoscopy is recommended [20]–[22]. In our study, colon cancer was found in five patients (10.6%) and was the second most frequent cancer.

IGF-1 is an important factor for replication of normal thyroid follicular cells and reducing apoptosis [23]. Increased IGF-1 stimulation may increase carcinogenesis and act with other initiating factors to promote progression of thyroid cancer from an occult to a clinically relevant stage [7], [24]. In a recent meta-analysis by Wolinski et al., thyroid cancer occur significantly more often in acromegalic patients than in general population (OD = 7.5, RR7.2) [6], and a recent case-control study showed that thyroid cancer has a 10.21 increased risk in patients with acromegaly compared with that in a control group [25]. In our study, the prevalence of PTC was 25% in patients with acromegaly, and uncontrolled acromegaly was significantly higher in frequency in the PTC group. Recent studies have reported that cancer incidence and cancer-related mortality rates are elevated in patients with persistent active disease [19], [26]. These results suggest that long-term stimulation by GH and IGF-1 from thyroid follicular cells may be responsible for thyroid carcinogenesis in patients with acromegaly.

IGF-1R is a hetero-tetrameric protein, consisting of two extracellular α-subunits that bind IGF and two transmembrane β-subunits bearing intrinsic tyrosine kinase activity [27]. IGF-1 binds IGF-1R and activates the phosphatidylinositol-3 kinase and AKT/protein kinase B pathways and their phosphorylation, which are anti-apoptotic mechanisms that also activate the RAS/MEK/MAPK pathway as a cellular differentiation, proliferation, senescence and survival mechanism [23], [28], [29]. IGF-1R is overexpressed in tumors from several anatomical sites, including normal and malignant thyroid follicular cells [24], [30]. Several clinical and experimental studies have reported that increased circulating IGF-1 levels and increased expression of IGF-1 and IGF-1R in tumor tissues are involved in the development of these malignant tumors [31]. In our study, IGF-1Rβ was expressed by 100% of thyroid cancer cells and was stained more strongly in cancer tissue than in adjacent normal tissue, which is similar to a previous report [32]. IGF-1Rβ IHC staining in normal thyroid tissue adjacent to cancer tissue was significantly less intense in acromegalic PTC compared with that in non-acromegalic PTC. Increased serum IGF-1 levels in patients with acromegaly may downregulate IGF-1Rβ; however, the autocrine and paracrine effects of IGF-1 can be induced by local expression of IGF-1Rβ in tumor tissue. This may partly contribute to the abnormal growth of tumors, and is recognized as an attractive target for cancer treatment.

PTCs frequently (up to 83% of PTCs in Korea) harbor an activating mutation of *BRAF* ^V600E^ [8]. The *BRAF* ^V600E^ mutation suggests that ERK, a downstream effector of *BRAF*, may play a major role in the carcinogenesis of PTC [33], and is associated with extrathyroid invasion, lymph node metastases, advanced tumor stage, and frequent recurrence [34]. Multi-kinase inhibitors such as sorafenib, which target vascular endothelial growth factor receptors 2 and 3, common *RET/PTC* subtypes, and *BRAF*, have shown great promise in the treatment of malignancies harboring a *BRAF* ^V600E^ mutation [35]. However, the *BRAF* ^V600E^ mutation is rare in acromegalic patients with PTC, and these patients should be treated with an anti-IGF-1R therapeutic approach [36].

Several limitations to our study should be mentioned. No control group for comparison of the prevalence of thyroid cancer in patients with acromegaly was included. The overall thyroid cancer prevalence is 76.9 and 427.5 per 100,000 in males and females, respectively in a 2011 study in Korea [18]. A second limitation regards a potential bias, because most of the thyroid cancers were microcarcinoma (58.3%). We performed FNAC for thyroid nodules suspicious of malignancy regardless of size, whereas nodules ≥1 cm and with suspicious US features were evaluated by FNAC in other studies [3], [25]. The prevalence of occult PTC at autopsy could be as high as 35% [37], and small occult PTCs (<5 mm in diameter) are considered not to require treatment [38]. Therefore, the high prevalence of thyroid cancer in our study may have been caused by active thyroid screening. Last, our findings are limited by the small sample size and the short follow-up period for examining cancer-related mortality or recurrence. In general, PTC is associated with a good prognosis. Some reports show that IGF-1R tumor expression is an aggressive clinical feature and persistent despite thyroid cancer treatment [39], . Further study should be conducted to determine the prognosis of patients with acromegaly.

In conclusion, the rate of thyroid cancer was extremely high (25%) in our study, and it was the most common cancer among our patients with acromegaly. Uncontrolled acromegaly implies that persistently elevated GH and IGF-1 levels may be present in patients with a high risk of developing thyroid cancer. Therefore, regular thyroid US screening and FNAC for all suspicious thyroid nodules should be considered in all patients with newly diagnosed acromegaly and poorly controlled disease. PTC that develops in patients with acromegaly may have a different prognosis or be treated with a different modality, because a hyperactive GH-IGF-1 axis may play a dominant role in development of PTC rather than the *BRAF* ^V600E^ mutation. Further studies on this subject are required, as this was a single-center, retrospective study with a small sample size.
